# Assessment of cognitive function in patients with schizophrenia based on virtual reality serious games: a prospective nonrandomized clinical trial

**DOI:** 10.3389/fpsyt.2025.1608905

**Published:** 2025-08-06

**Authors:** Chenxin Wu, Yu Zhuo, Jianying Yu, Xingxing Li, Wenting Zhao, Xiandong Meng

**Affiliations:** ^1^ Mental Health Center, West China Hospital, Sichuan University/West China School of Nursing, Sichuan University, Chengdu, China; ^2^ West China Hospital, Sichuan University, Chengdu, China; ^3^ West China School of Public Health and West China Fourth Hospital, Sichuan University, Chengdu, China

**Keywords:** virtual reality, serious game, schizophrenia, cognitive assessment, ecological validity, diagnostic validity

## Abstract

**Introduction:**

Conventional neuropsychological tests for assessing cognitive function in schizophrenia face critical limitations in ecological validity and efficiency. We developed *Fruit Pioneer*, a virtual reality serious game simulating real-world cognitive demands, to address these challenges. This study aimed to validate its diagnostic validity in differentiating cognitive impairments between schizophrenia patients (SZs) and healthy controls (HCs), while evaluating user experience feasibility.

**Methods:**

In this cross-sectional study, 107 participants (43 SZs, 64 HCs) underwent cognitive evaluation using the Brief Cognitive Assessment Tool for Schizophrenia (B-CATS) and *Fruit Pioneer*. Diagnostic validity was analyzed via receiver operating characteristic (ROC) curves and Spearman correlations. User experience was quantified using the Game Experience Questionnaire Core Module (GEQ-Core) and Simulator Sickness Questionnaire (SSQ).

**Results:**

SZs exhibited significantly poorer performance on B-CATS compared to HCs (all subtests *p* < 0.05). Among 16 *Fruit Pioneer* performance indicators, 13 demonstrated strong discriminative power (AUC > 0.7). The Total Game Score (TGS) achieved high accuracy (AUC = 0.911, sensitivity = 83.72%, specificity = 89.06%). Directionally consistent correlations were observed between game indicators and B-CATS scores (e.g., TGS vs. Digit Symbol Substitution Test: *r* = 0.66, *p* < 0.01). Participants reported high immersion (GEQ-Core immersion: 2.45/4) and minimal simulator sickness (SSQ total: 5.12/48).

**Conclusion:**

*Fruit Pioneer* provides a time-efficient (5-minute), ecologically valid tool for cognitive assessment in schizophrenia, demonstrating strong discriminative validity and user acceptability. Further validation should explore its clinical utility in broader populations and the association between its performance and functional outcomes.

## 1 Introduction

Schizophrenia is a severe psychiatric disorder that affects approximately 24 million individuals globally (1). This condition is associated with considerable disability and may affect all areas of life including personal, family, social, educational, and occupational functioning (2). Cognitive impairment is one of the core symptoms of schizophrenia (3). There are seven major dimensions for domains of cognitive deficits in schizophrenia: Speed of Processing, Attention/Vigilance, Working Memory, Verbal Learning and Memory, Visual Learning and Memory, Reasoning and Problem Solving, and Social Cognition (4). These deficits span the course of the disease, starting from the prodrome, and remain stable over time (5). The longitudinal relationships between cognitive performance and functional outcomes in schizophrenia have been well documented (6). Persistent cognitive deficits have a substantial impact on the outcome of schizophrenia, and are often associated with poor quality of life (5, 7). In order to enhance functional outcomes and quality of life in schizophrenia, early assessment and treatment of cognitive impairment in individuals with schizophrenia would be significant.

Assessment of cognition in patients with schizophrenia would help clinicians and families anticipate the degree of problems and success in work, school, social functioning, or rehabilitation (8). Furthermore, the clinical development of new treatments also demonstrates a reliance on cognitive assessment (9, 10). Neuropsychological testing is the gold standard for the assessment of cognitive functioning (11). The majority of studies used objective neuropsychological test batteries to measure cognitive impairments in schizophrenia. These tests were well-established, and the rigor of these testing procedures ensures standardized results, ensuring coherent interpretations across studies (12). However, traditional neuropsychological tests also have important limitations:(1) Weak ecological validity: These tests are administered in controlled environments with rigid instructions, bearing minimal resemblance to real-life cognitive challenges (13–15). Patients typically perform isolated tasks targeting single cognitive domains, whereas real-world scenarios demand multitasking (16), limiting generalizability to daily functioning. (2) Low engagement: Paper-and-pencil or computer-based formats (17), lack immersive engagement, increasing susceptibility to external distractions. Performance is further influenced by age, language proficiency, and literacy levels (18). (3) Time and expertise barriers: Most tests are time-intensive (typically 60–90 minutes) and require administration by trained professionals to ensure standardization (19), These factors—prolonged duration and reliance on specialized staff—hinder their utility in resource-constrained clinical settings (4) Hawthorne effects: Anxiety induced by overt observation may alter participants’ behaviors, compromising the authenticity of cognitive performance (20). These limitations underscore the need for novel tools that assess cognition with ecological relevance, high engagement, and efficiency.

Virtual reality (VR) is defined as a computer-generated simulation, including images and sounds that represent real places or situations. Users interact with these simulations using special electronic equipment, experiencing them in a seemingly real or physical way. Through a headset, it transmits visual, auditory, and various sensations to users, making them feel as if they are in a virtual or imagined environment (21). VR platforms enable simulation of immersive, engaging, naturalistic cognitive challenges while maintaining a controlled experimental environment. Virtual reality is therefore a promising assessment modality for ecologically valid measurement of cognitive function (14, 22). To maintain and increase participant’s involvement, process of cognitive assessment needs to be interesting and attractive. Serious games can fulfill this requirement. Serious games are digital games created with the intention to entertain and to achieve at least one additional goal (e.g., learning or health) (23), with an increasing application in healthcare (24).These tools demonstrate versatility across neuropsychiatric conditions: In ADHD, ClinicaVR: Classroom-CPT provides ecologically valid attention assessments by simulating classroom distractions, improving diagnostic accuracy compared to traditional continuous performance tests (25); in cognitive screening, CAVIRE reduces assessment time by 1–2 minutes compared to MoCA while maintaining test consistency across age groups, demonstrating VR’s efficiency advantage (18).

In recent years, VR-based training programs and serious games have been increasingly used to improve and assess cognitive function in patients with cognitive impairments. They provide an immersive environment that simulates real-world scenarios, enhancing their effectiveness for cognitive rehabilitation and assessment. For example, Miskowiak et al. developed the first immersive virtual reality cognition assessment tool, the Cognition Assessment in Virtual Reality (CAVIR), which requires participants to complete five sub-tasks in a kitchen scenario assessing verbal memory, processing speed, attention, working memory, and planning skills (14). Similarly, Borghetti et al. explored the use of the Enhance VR app, based on validated neuropsychological principles, offered a more comprehensive and realistic assessment of cognitive function (26). However, while both tools demonstrate sensitivity to cognitive impairments, their reliance on a multi-step narrative context may increase cognitive load and administration time. This could potentially limit their feasibility for populations with lower educational attainment or acute symptoms.

To address these limitations, we developed *Fruit Pioneer*—a VR serious game that eliminates complex narratives and contextual backgrounds, adopts a language-agnostic design using universally recognizable objects (e.g., fruits), and delivers time-efficient assessment within 5 minutes. Targeting schizophrenia-specific cognitive deficits, this system grounds interactions in ecologically valid scenarios with everyday items, providing an engaging tool for clinical implementation. The objectives of this study were to (1) examine the diagnostic validity of the virtual reality serious game *Fruit Pioneer* in differentiating individuals with cognitive impairments from healthy controls, and (2) evaluate the user experience of *Fruit Pioneer* as a cognitive assessment tool.

## 2 Methods

### 2.1 Participants

The trial was conducted in accordance with the Declaration of Helsinki, and has been approved by the Medical Research Ethics Committee of Sichuan University (Reference number: 2023-1926) All participants signed written informed consent forms. Individuals were enrolled at Mental Health Center of West China Hospital, Sichuan University, from May to December 2023.Inclusion criteria for SZs included: (1) aged 18 to 40 years; (2) hospitalized with ICD-10 schizophrenia diagnosis and in clinical stability phase, defined as effective control of acute positive symptoms (e.g., hallucinations, delusions, thought disorders, behavioral disturbances) confirmed by treating psychiatrist; (3) No acute symptom exacerbation (validated low-risk scores for suicide, aggression, and elopement); (4) No imminent pharmacotherapy adjustments planned. Exclusion criteria included: (1) colour blindness; (2) visual impairment (requiring glasses for correction); (3) substance abuse (including alcohol dependence); (4) comorbid chronic physical diseases. Inclusion criteria for HCs were: (1) aged 18 to 40 years; (2) healthy individuals. Exclusion criteria were the same as for SZs.

### 2.2 Materials

#### 2.2.1 Fruit Pioneer

**Figure 1:**
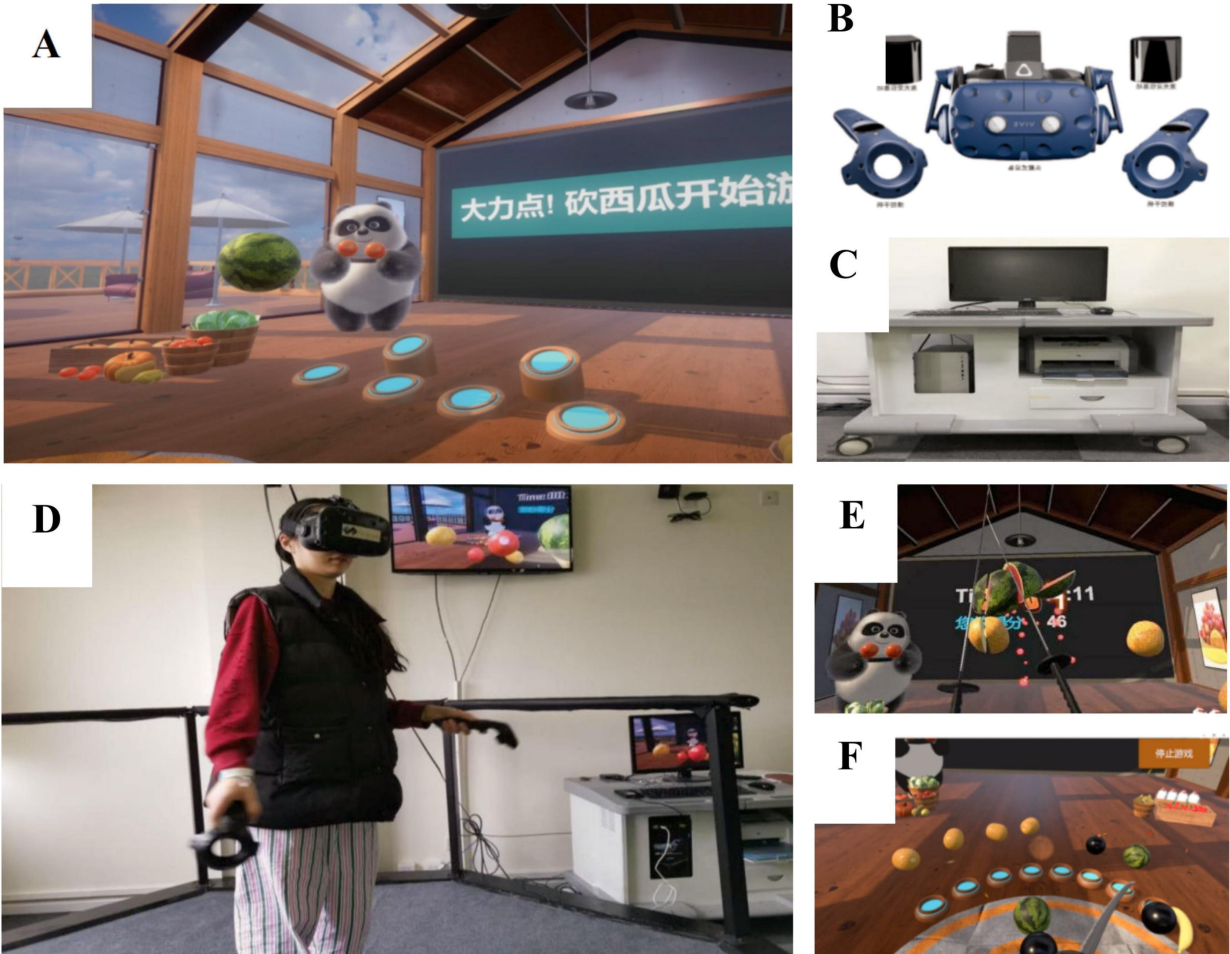
*Fruit Pioneer*: **(A)** *Fruit Pioneer* environment; **(B)** VR head-mounted display and headphones, handles; **(C)** Personal computer; **(D)** Participants are playing the game; **(E)** Users’ field of vision: a swinging panda, a scoreboard, fruits and two long knives; **(F)** Users’ field of vision: a circle of fruit-shooting spouts.

*Fruit Pioneer* is a self-designed VR serious game adapted from popular fruit-cutting games, aimed at assessing and improving cognitive function in individuals with schizophrenia. People diagnosed with schizophrenia generally have impaired cognitive function and reduced ability to deal with complex situations. Consequently, the game design avoids complex story backgrounds and tasks, focusing on five common types of fruits as the primary cutting targets. Players score points for each successful cut, while bombs serve as inhibitory factors that result in score deductions if hit. The objective is to cut as many fruits as possible while avoiding bombs. The key design elements of the fruits and bombs are as follows: Fruit Types: The game features bananas, apples, watermelons, oranges, and pineapples, which are common in daily life and vary in colour, shape, and size. Fruit Colours: The colours of the fruits in the game are consistent with their real-life counterparts. Remembering the colour-target object match helps quickly identify the cutting target and score higher. However, patients with schizophrenia often have memory impairments, so reinforcing the connection between colours and targets through repetition can train their memory function. Fruit Shapes: The fruits have different shapes, affecting cutting difficulty. Bananas are elongated, and cutting them from the cross-section is relatively easier. Fruit Sizes: Different fruit sizes influence cutting difficulty. Smaller fruits are more challenging to visually track and hit accurately, thus they are assigned higher points. Larger fruits are more visually stimulating and easier to perceive, making them easier targets and thus assigned lower points. Points vary based on size: apples, oranges, and pineapples score 3 points each, bananas score 2 points each, and watermelons score 1 point each. This scoring system encourages players to prioritize cutting smaller fruits for higher scores within a limited time, which requires the use of executive function. Patients with schizophrenia often have impaired executive function and may prioritize the first target they see rather than making optimal decisions. Additionally, patients are more likely to notice larger targets due to visual system impairments, leading them to prefer cutting larger fruits during the game. Fruit Motion: Fruits move in straight lines, making it easier for players to track and predict their positions for accurate cutting, requiring decision-making and cognitive function engagement. The random appearance of fruits in the game space prevents players from developing passive cutting behaviours after memorizing fixed positions of different fruits. Bomb Design: Bombs are included as inhibitory factors. Hitting a bomb results in a 5-point penalty. Bombs are designed to minimize interference by resembling real-life hand grenades in size. Given that patients with schizophrenia often dislike the colour black, bombs are black to reduce the likelihood of accidental hits. The black colour contrasts with the fruits, making bombs easier to identify and avoid. Since patients have difficulties recognizing similar shapes, the bombs are round to match the round shapes of four types of fruits, maintaining the game’s difficulty (see **Figure 1**).

The software was developed using Unity3D (version 2018.3.0f2, Unity Technologies) and runs on a personal computer (HP PC, Intel Core i5-9400F processor, 16 GB DDR4–3000 MHz memory, GTX 1660 6GB graphics card, 256 GB solid-state drive, and 1 TB hard disk drive) and the HTC Vive (HTC Corporation) VR head-mounted display. During the game, participants wear the head-mounted display and hold the handles. Headphones of the VR goggles play joyful game sounds that change with the content and pace of the game. Users hold a handle device in each hand, and the device is instantly connected to the VR goggles when the bottom is pressed, visually changing into two long knives. The game is played from a first-person perspective, with a swinging panda, a scoreboard, and a circle of fruit-shooting spouts in the user’s field of vision. Fruits may be shot from any of the spouts, so users are expected to turn around and search for fruits. The study includes 16 performance indicators on *Fruit Pioneer* (see **Table 1**).

**Table 1:** Performance indicators on *Fruit Pioneer*.

Number	Indicator	Description
1	BAN	Hits of bananas
2	APP	Hits of apples
3	WM	Hits of watermelons
4	OR	Hits of oranges
5	PIN	Hits of pineapples
6	BOMB	Hits of bombs
7	TGS	Total game score
8	TFS	Total fruit score
9	TH	Total hits^*^
10	THF	Total hits on fruits
11	TMF	Total misses on fruits
12	SFH	Hits on small fruits^*^
13	PSH	Proportion of hits on small fruits in total hits
14	PSF	Proportion of hits on small fruits in total fruit hits
15	PSS	Proportion of scores from hits on small fruits in total score
16	PSSF	Proportion of scores from hits on small fruits in total fruit score

Total hits (TH) include fruits and bombs; Hits on small fruits (SFH) include apples, oranges, and pineapples.

#### 2.2.2 Brief Cognitive Assessment Tool for Schizophrenia

We evaluated cognitive function using the Brief Cognitive Assessment Tool for Schizophrenia (B-CATS) developed by Hurford IM et al (27, 28). The B-CATS is a simple instrument to measure cognitive function that generally takes 12 min to finish. It contained 4 subtests, namely, the Digital Symbol Substitution Test (DSST), Trail Making Test part A (TMTA), Trail Making Test part B (TMTB), and Animal Fluency (AF). In the DSST, participants were presented with a sheet having 9 symbols paired with digits 1–9 on the top and rows of symbols beneath. Participants were asked to pair each symbol with its corresponding digit within 120 s. The number of correct pairs was the score. There were 120 symbols on the sheet, the first 10 of which were used for examples. Therefore, the maximum score for this test was 110. The DSST reflected the participant’s work memory. TMTA and TMTB demanded that participants draw a “trail” in numerical order of numbers or from number to letter (1-A-2-B) without taking the pen off the sheet. Participants were stopped and returned to the last correct response when they made a mistake. Time to completion was the score. TMT indicated the participant’s executive function. In the AF part, participants were given 60 s to name as many animals as possible. The score was the number of animals named. AF measures verbal fluency associated with social cognition. The test-retest reliability of each subtest of the scale ranges from 0.61 to 0.73, and the correlation with the gold standard for schizophrenia cognitive function assessment, the MATRICS Consensus Cognitive Battery (MCCB), is 0.76. The scale demonstrates good psychometric properties and advantages in terms of shorter administration time and ease of implementation compared to traditional schizophrenia cognitive function assessment tools.

#### 2.2.3 Game Experience Questionnaire Core Module

We evaluated game experience using the Game Experience Questionnaire Core Module (GEQ-Core Module), developed by IJsselsteijn et al. The questionnaire encompasses essential aspects of game experience assessment, containing 7 dimensions and 33 items. Each dimension consists of specific items: Competence dimension includes items 2, 10, 15, 17, and 21; Immersion dimension includes items 3, 12, 18, 19, 27, and 30; Flow dimension includes items 5, 13, 25, 28, and 31; Challenge dimension includes items 11, 23, 26, 32, and 33; Positive Affect dimension includes items 1, 4, 6, 14, and 20; Negative Affect dimension includes items 7, 8, 9, and 16; Tension/Annoyance dimension includes items 22, 24, and 29. The questionnaire employs a Likert 5-point rating scale: 0 indicates ‘not at all’, 1 indicates ‘slightly’, 2 indicates ‘moderately’, 3 indicates ‘very much’, and 4 indicates ‘extremely’. Differences in participants’ experiences across various dimensions are evaluated by computing the average scores for each dimension (29).

#### 2.2.4 Simulator Sickness Questionnaire

We evaluated simulator sickness and unwanted negative side effects following immersions in VR serious game using the Simulator Sickness Questionnaire (SSQ) developed by Kennedy et al. The questionnaire had 16 items divided into two dimensions. Each dimension and its items were nausea (items 1, 6, 7, 8, 12, 13, 14, 15, 16) and oculomotor nerve symptoms (items 2, 3, 4, 5, 9, 10, 11). Participants rated the severity of each symptom on a 4point Likert scale (0 – “None” to 3 – “Severe”). To obtain a total score, all raw items were summed. A higher total score reflects more severe sickness. The Cronbach’s Alpha for the entire scale was 0.87 (30).

### 2.3 Procedure

For all participants, both SZs and HCs, the experimental procedures consisted of three sequential stages conducted under the supervision of trained medical staff.

First, cognitive assessment was performed using the Brief Cognitive Assessment Tool for Schizophrenia (B-CATS).

Second, the VR serious game session was administered following a standardized protocol: Prior to gameplay, clinical safety screening confirmed participants’ symptom stability (absence of active psychosis such as hallucinations or delusions); researchers then provided detailed equipment familiarization and rule instruction, including proper handling of VR controllers; this was followed by a 5-minute practice trial to ensure task comprehension; after confirmation of readiness, participants completed the formal 5-minute Fruit Pioneer session under continuous clinician monitoring, with immediate termination upon request or observed distress.

Third, post-session evaluation included completion of the Game Experience Questionnaire Core Module (GEQ-Core) and Simulator Sickness Questionnaire (SSQ), along with brief symptom assessment by the treating psychiatrist.

Throughout all stages, professional medical staff ensured participant safety, accurate data collection, and protocol adherence. Psychiatric evaluations post-session confirmed no emergent symptom exacerbation. All participants completed these tasks in the standardized sequence without deviations, and no withdrawals occurred due to VR-related adverse effects.

### 2.4 Safety consideration

Literature review indicates that VR applications in schizophrenia research, assessment, and treatment demonstrate robust safety profiles, with minimal to no intervention-related adverse effects (31, 32). VR interventions show positive therapeutic effects across core psychotic symptoms, including positive symptoms and delusions (33, 34).

In this study, the VR gameplay session implemented evidence-based safety protocols to address psychotic symptoms: Pre-session psychiatric screening excluded active psychosis; equipment familiarization used neutral demo scenes; and strictly timed 5-minute gameplay employed non-provocative stimuli (fruit/bomb objects without social narratives) to prevent symptom exacerbation. Sessions were immediately terminated upon observed distress (no instances occurred).

### 2.5 Statistical analysis

The statistical analyses were performed using IBM-SPSS statistics (Version 26) and R language (Version 4.3.3), a two-tailed p-value <0.05 was considered as statistically significant. For categorical variable, frequencies and proportions were used to describe the characteristics of the participants, Chi-square was used for comparison. For continuous variables, the mean plus or minus the standard deviation (M ± SD) were used to describe the study variables with a normal distribution, medians with the interquartile range (IQR) were used to describe the study variables with non-normal distribution. The Shapiro–Wilk test was used for normality testing. An independent t test was used to compare the data with a normal distribution. If the data did not obey a normal distribution, the Mann–Whitney U test was used for comparison. Additionally, spearman correlations were used to assess the correlations between study variables. The receiver operating characteristic (ROC) curve was plotted and the area under the curve (AUC) was calculated to assess the predictive effects of performance indicators on *Fruit Pioneer* for cognitive impairment. Sensitivity and specificity were calculated to evaluate the validity of indicators; the predictive value (PPV and NPV) and likelihood ratio (LR+, LR-) were used to evaluate the efficacy of indicators. Youden’s index was calculated as “sensitivity + specificity −1”, and the score for which the Youden’s index was maximal was set as the optimal cut-off value for each indicator.

## 3 Results

### 3.1 Demographic characteristics of SZs and HCs

In this study, 107 participants were finally included, 40.2%(n=43) were SZs, 59.8%(n=64) were HCs. The demographic data of the participants in the two groups were compared. There was no significant difference in sex between SZs and HCs(p=0.723). but significant differences were observed in age(p<0.01) (see **Table 2**).

**Table 2:** Comparison of demographic characteristics between patients with schizophrenia and healthy controls.

Variables		[M (Q1, Q3)]/%		*Z/χ^2^*	*p*	Effect size (r)
		SZs(n=43)	HCs(n=64)			
Gender ^a^	Male	27(62.8%)	38(59.4%)	0.126	0.723	
	Female	16(37.2%)	26(40.6%)			
Age (year) ^b^		25(19,28)	20(20,21)	-3.207	<0.01^**^	0.31

^a^ Means that the difference was assessed by Chi-square; ^b^ Means that the difference was assessed by Mann–Whitney U test; ^**^ *p*<0.01.

[M (Q1, Q3)], [median (first quartile, third quartile)]; SZs, patients with schizophrenia; HCs, healthy controls.

### 3.2 Cognitive function of SZs and HCs

The statistical results indicated that there was no significant difference in sex between SZs and HCs, but significant differences were observed in age. To avoid the interference of confounding factors on B-CATS test scores, we included age as adjusting factors and corrected the B-CATS test scores for both groups.

Before comparing the cognitive function of SZs and HCs, the Shapiro–Wilk test was performed on B-CATS scores. The results showed that the DSST (*p*1 = 0.340, *p*2 = 0.041) and AF (*p*1 = 0.167, *p*2 = 0.411) scores nearly obeyed a normal distribution, while the TMTA (*p*1 <0.05, *p*2 <0.05) and TMTB (*p*1 <0.05, *p*2 <0.05) scores did not. Therefore, an independent t test was used to compare the DSST and AF, and the Mann–Whitney U test was used to compare the TMTA and TMTB between the two groups. There were significant differences between the two groups in scores of the DSST, TMTA, TMTB, and AF, the scores in SZs were significantly lower than those in HCs (see **Table 3**).

**Table 3:** Comparison of B-CATS scores between patients with schizophrenia and healthy controls.

B-CATS	Group	(Mean ± SD)	Z-Score	*p*	95% CI	Cohen's d/r
DSST ^a^	SZs(n=43)	23.87±21.84	-2.698	<0.01^**^	-57.916~-41.784	2.51
	HCs(n=64)	73.72±18.48				
TMTA ^b^	SZs(n=43)	80.63±56.43	5.927	<0.01^**^	51.561~86.731	0.73
	HCs(n=64)	11.48±11.67				
TMTB ^b^	SZs(n=43)	204.6±152.35	3.381	<0.01^**^	134.090~231.325	0.68
	HCs(n=64)	21.89±54.05				
AF ^a^	SZs(n=43)	11.38±11.58	-1.408	<0.01^**^	-21.678~-12.380	1.44
	HCs(n=64)	28.40±12.09				

^a^ Means that the difference was assessed by independent t test; ^b^ Means that the difference was assessed by Mann–Whitney U test; ^**^ *p*<0.01.

(Mean ± SD), (mean ± standard deviation); SZs, patients with schizophrenia; HCs, healthy controls; DSST, digital symbol substitution test; TMTA, trail making test part A; TMTB, trail making test part B; AF, animal fluency.

### 3.3 Performance of the VR serious game *Fruit Pioneer*

#### 3.3.1 Comparison of Performance on *Fruit Pioneer* between SZs and HCs

**Figure 2:**
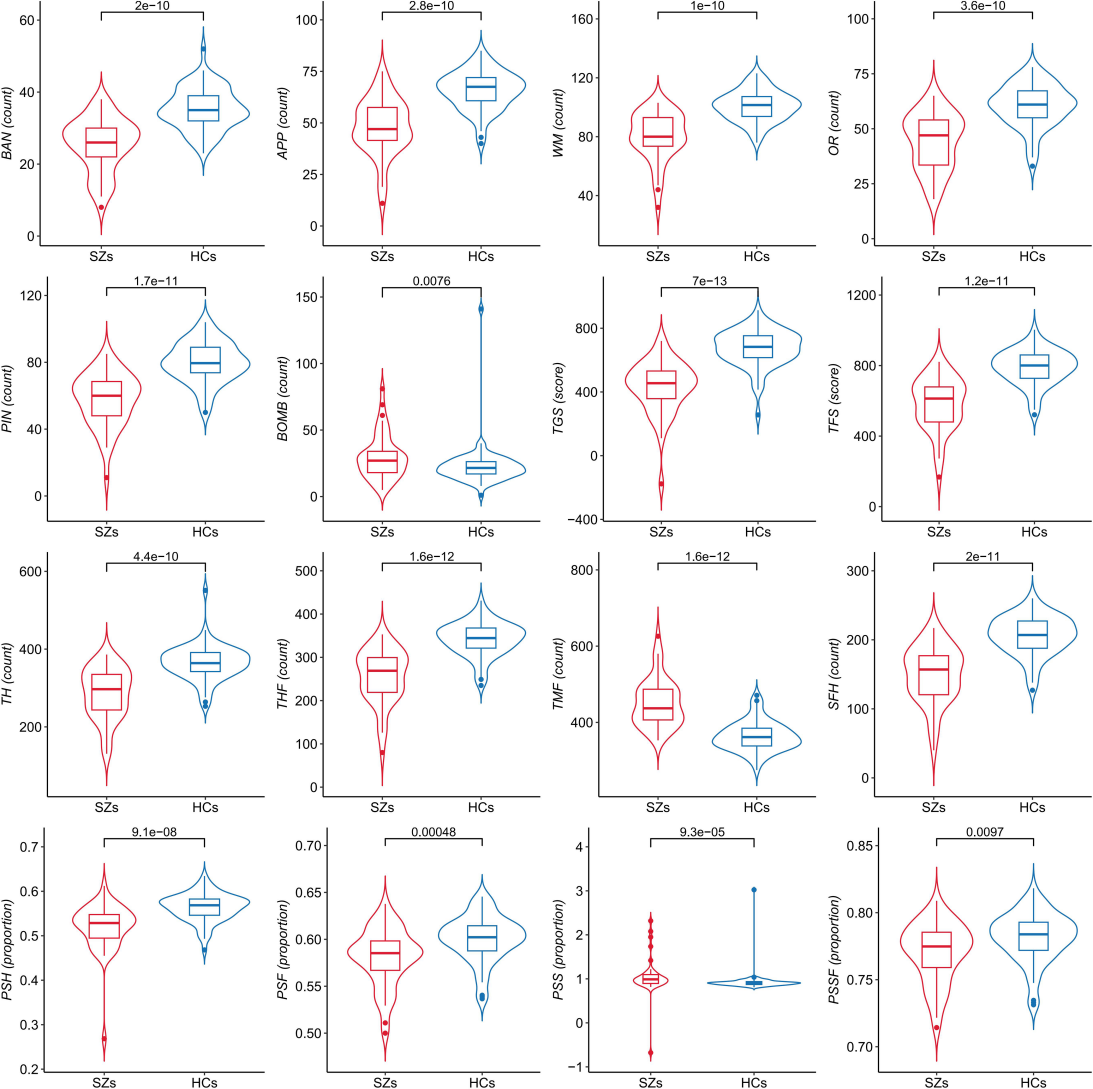
Comparison of performance indicators on *Fruit Pioneer* between patients with schizophrenia (SZs) and healthy controls (HCs).

As shown in **Figure 2**, we compared differences in 16 performance indicators on *Fruit Pioneer* between SZs (n = 43) and HCs (n = 64). The violin plots show the distribution of each variable, the inner boxplots represent summary statistics. For indicators with normally distributed data, we used the two-sample t-test, and for those with non-normally distributed data, we used the Mann-Whitney U test. The results showed that there were significant differences between the two groups in 16 indicators (all p <0.05), and the specific P values are shown in the figure.

#### 3.3.2 Correlations of Performance on *Fruit Pioneer* with B-CATS

**Figure 3:**
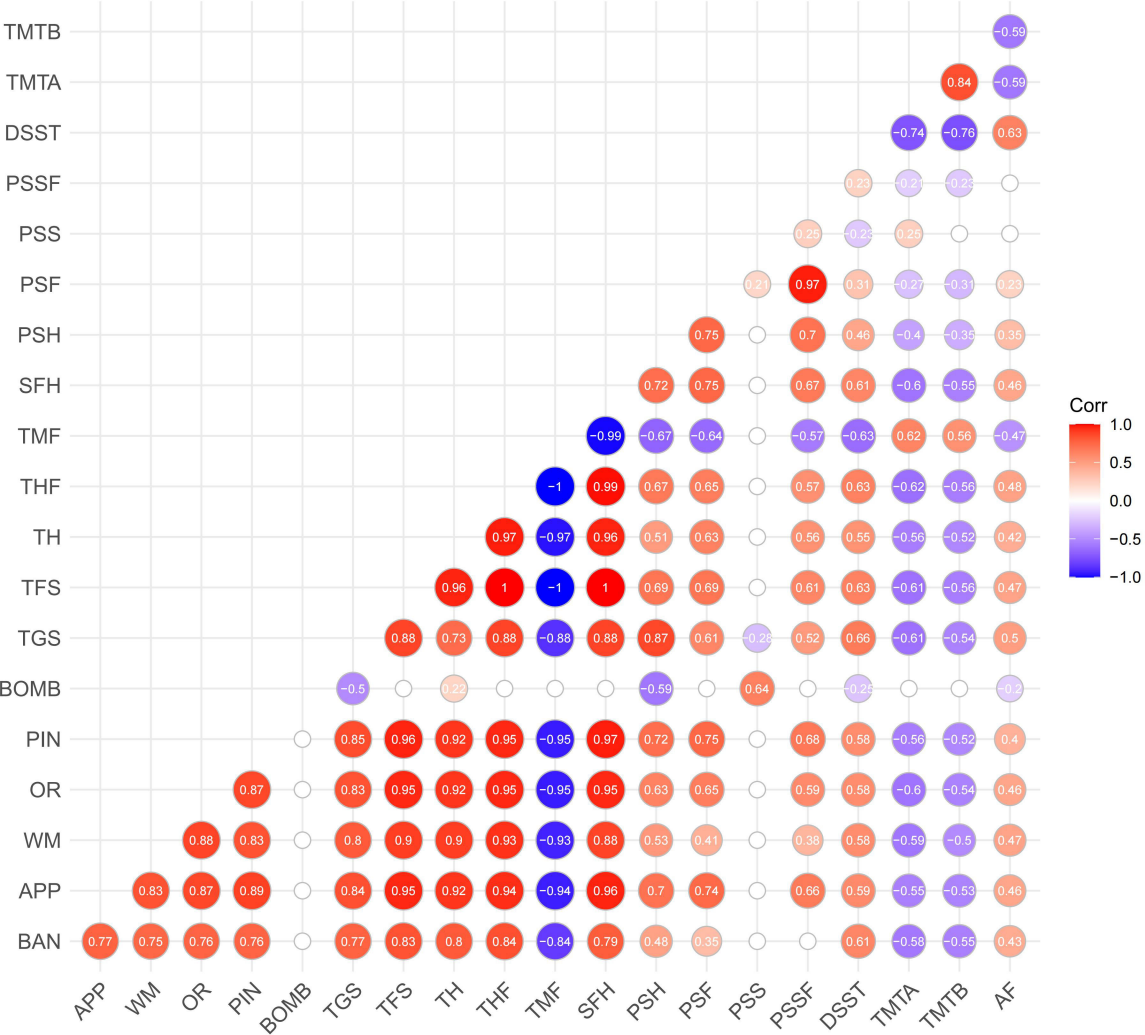
Correlation matrix of performance indicators on *Fruit Pioneer* and B-CATS scores.

The correlation matrix (see **Figure 3**) shows the relationship between the performance indicators on *Fruit Pioneer* (BAN to PSSF) and B-CATS scores (including DSST, TMTA, TMTB, AF). Most performance indicators on *Fruit Pioneer* exhibit significant positive correlations with each other, especially among the indicators related to the number of fruit hits, total game score, and total fruit score (e.g., TGS, TFS, THF, TH). Strong positive correlation indicates that these indicators may play common role in the evaluation of cognitive function. The number of bomb hits (BOMB) shows negative correlations with most other indicators, particularly with the total game score (TGS) and total fruit score (TFS), where the negative correlations are the most pronounced. The negative correlation between BOMB and the other indicators that hitting bomb significantly decreases player performance, which is consistent with the inhibitory effect of game design.

Although the correlation between the B-CATS scores and performance indicators is weak, but still shows some meaningful trend. Several performance indicators (TGS, TFS, TH, THF, TMF, SFH, PSH, PSF) show consistent correlations with B-CATS scores (DSST, TMTA, TMTB, AF). Specifically, indicators where higher numerical values indicate better performance (TGS, TFS, TH, THF, SFH, PSH, PSF) are positively correlated with DSST and AF (where higher scores also indicate better performance) and negatively correlated with TMTA and TMTB (where higher scores indicate poorer performance). Conversely, TMF (where higher numerical values indicate poorer performance) is negatively correlated with DSST and AF, and positively correlated with TMTA and TMTB. This suggests that participants’ performance on *Fruit Pioneer* is somewhat consistent with their performance on the B-CATS tests.

#### 3.3.3 Diagnostic validity of performance indicators on *Fruit Pioneer*

**Figure 4:**
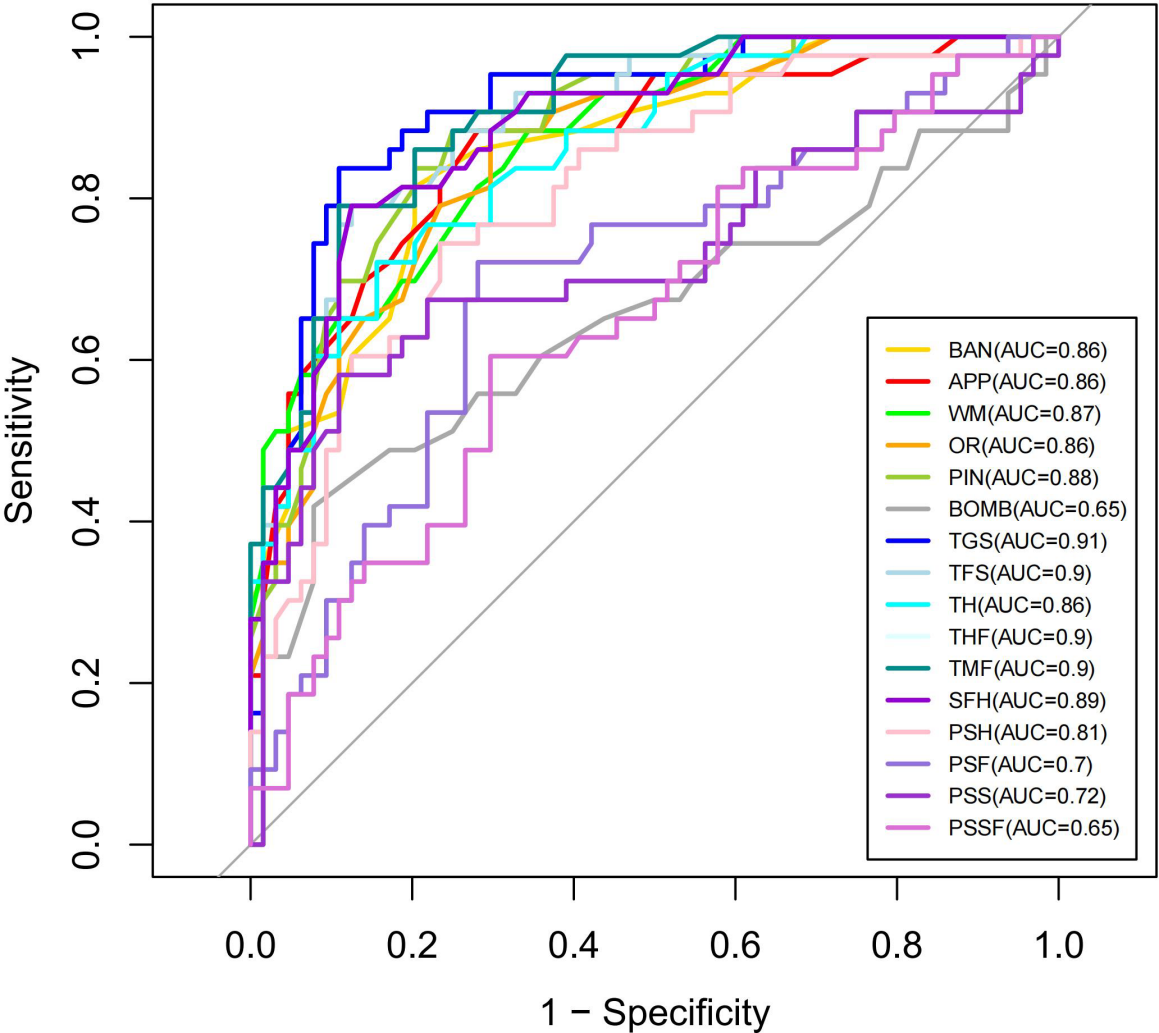
ROC curves of performance indicators on *Fruit Pioneer*.

The ROC curves for performance indicators of the VR serious game *Fruit Pioneer* (BAN to PSSF) are shown in **Figure 4**. The AUCs for the majority of indicators were above 0.7, indicating good efficacy in distinguishing between individuals with schizophrenia and healthy controls. The five indicators with the highest AUC value are TGS (AUC 0.911, 95% CI 0.855-0.967, sensitivity 83.72%, specificity 89.06%), THF (AUC 0.904, 95% CI 0.849-0.959, sensitivity 79.07%, specificity 89.06%), TMF (AUC 0.904, 95% CI 0.849-0.959, sensitivity 79.07%, specificity 89.06%), TFS (AUC 0.896, 95% CI 0.838-0.954, sensitivity 79.07%, specificity 87.5%), SFH (AUC 0.887, 95% CI 0.825-0.949, sensitivity 79.07%, specificity 87.5%), demonstrating the strongest discriminative ability. Conversely, the AUC values for PSSF (AUC 0.648, 95%CI 0.541-0.755, sensitivity 60.47%, specificity 70.31%), BOMB (AUC 0.653, 95%CI 0.538-0.767, sensitivity 41.86%, specificity 92.19%), PSF (AUC 0.69985, 95%CI 0.596-0.803, sensitivity 72.09%, specificity 71.88%) are below 0.7, indicating weaker discriminative ability. AUC, 95%CI, Cut-off point, Sensitivity, Specificity, PPV (positive predictive value) NPV (negative predictive value), LR+ (positive likelihood ratio), LR- (negative likelihood ratio) of 16 indicators of the VR serious game *Fruit Pioneer* (BAN to PSSF) are shown in **Table 4**.

**Table 4:** ROC results of performance of the VR serious game *Fruit Pioneer*.

	AUC (95%CI)	Cut-off point	Sensitivity (%)	Specificity (%)	PPV (%)	NPV (%)	LR+	LR-
BAN	0.858(0.788-0.929)	<31.5	81.40	79.69	72.92	86.44	4.01	0.23
APP	0.864(0.792-0.936)	<61.5	88.37	71.88	67.86	90.2	3.14	0.16
WM	0.870(0.804-0.935)	<89	65.12	89.06	80.00	79.17	5.95	0.39
OR	0.856(0.786-0.926)	<56.5	88.37	70.31	66.67	90.00	2.98	0.17
PIN	0.883(0.820-0.946)	<71.5	83.72	79.69	73.47	87.93	4.12	0.20
BOMB	0.653(0.538-0.767)	>30.5	41.86	92.19	78.26	70.24	5.36	0.63
TGS	0.911(0.855-0.967)	<569.5	83.72	89.06	83.72	89.06	7.65	0.18
TFS	0.896(0.838-0.954)	<690.5	79.07	87.50	80.95	86.15	6.33	0.24
TH	0.857(0.787-0.927)	<330.5	72.09	84.38	75.61	81.82	4.61	0.33
THF	0.904(0.849-0.959)	<302	79.07	89.06	82.93	86.36	7.23	0.24
TMF	0.904(0.849-0.959)	>404	79.07	89.06	82.93	86.36	7.23	0.24
SFH	0.887(0.825-0.949)	<177.5	79.07	87.50	80.95	86.15	6.33	0.24
PSH	0.806(0.721-0.890)	<54.53%	74.42	76.56	68.09	81.67	3.18	0.33
PSF	0.700(0.596-0.803)	<59.40%	72.09	71.88	63.27	79.31	2.56	0.39
PSS	0.724(0.616-0.832)	>98.31%	58.14	89.06	78.13	76.00	5.32	0.47
PSSF	0.648(0.541-0.755)	<77.66%	60.47	70.31	57.78	72.58	2.04	0.56

AUC, area under the curve; PPV, positive predictive value; NPV, negative predictive value; LR+, positive likelihood ratio; LR-, negative likelihood ratio.

### 3.4 Game experience and simulator sickness of *Fruit Pioneer*

After each participant’s VR gaming session, we used the Game Experience Questionnaire Core Module (GEQ-Core Module) and the Simulator Sickness Questionnaire (SSQ) to assess participants’ gaming experiences and simulator sickness. We analysed the scores of the GEQ-Core Module and SSQ scales for all participants, SZs, and HCs using descriptive statistics and compared the scores between SZs and HCs. For all participants, we found that the scores of the positive dimensions in the GEQ-Core Module for the VR game *Fruit Pioneer* were relatively high, while the scores of the negative dimensions were relatively low. Compared to HCs, SZs perceived lower levels of positive affect and higher levels of negative affect and tension, with statistically significant differences. For all participants, there was less simulator sickness in the VR game *Fruit Pioneer*. However, SZs experienced higher levels of oculomotor symptoms and overall simulator sickness compared to HCs, with statistically significant differences. The results are shown in **Table 5**.

**Table 5:** Game experience and simulator sickness of the VR serious game *Fruit Pioneer*.

Variables	Overall	SZs	HCs	t/Z	*p*	Cohen's d/r
Immersion ^b^	2.45±1.02	2.28±1.11	2.57±0.95	1.366	0.172	0.13
Flow ^a^	2.25±0.93	2.11±1.07	2.35±0.83	-1.244	0.217	0.25
Competence ^a^	2.25±0.87	2.10±0.93	2.36±0.82	-1.487	0.140	0.30
Positive ^b^	2.81±0.93	2.51±1.04	3.02±0.79	2.469	0.014^*^	0.24
Challenge ^aΔ^	1.31±0.72	1.36±0.84	1.27±0.64	0.623	0.535	0.12
Negative ^bΔ^	0.55±0.69	0.76±0.85	0.41±0.51	-2.440	0.015^*^	0.24
Tension ^bΔ^	0.46±0.69	0.66±0.85	0.33±0.52	-2.307	0.021^*^	0.22
Nausea ^bΔ^	2.66±3.58	3.53±4.50	2.08±2.67	-1.651	0.099	0.16
Oculomotor ^bΔ^	2.46±3.15	3.33±3.62	1.88±2.67	-2.656	0.008^**^	0.26
SSQ ^bΔ^	5.12±6.42	6.86±7.73	3.95±5.11	-2.275	0.023^*^	0.22

^a^ Means that the difference was assessed by independent t test; ^b^ Means that the difference was assessed by Mann–Whitney U test; ^*^ *p*<0.05; ^**^ *p*<0.01; ^Δ^ the higher the score, the worse the experience or discomfort.

SZs, patients with schizophrenia; HCs, healthy controls.

## 4 Discussion

To this study developed *Fruit Pioneer*, a virtual reality-based serious game designed to enhance ecological validity and clinical feasibility in cognitive assessment through simplified task contexts, decontextualized visual elements such as fruits and bombs, and a rapid 5-minute evaluation protocol. Correlation analyses with traditional neuropsychological tests (B-CATS), ROC curve validation of diagnostic validity, and user experience questionnaires—including the Game Experience Questionnaire Core Module (GEQ-Core) and Simulator Sickness Questionnaire (SSQ)—revealed three core findings: first, directionally consistent correlations between game performance indicators and B-CATS scores; second, high diagnostic validity, with the composite indicator Total Game Score (TGS) achieving an AUC of 0.911, sensitivity of 83.72%, and specificity of 89.06%, alongside 13 of 16 indicators demonstrating AUC values exceeding 0.7; third, enhanced clinical feasibility, characterized by an average assessment duration of 5 minutes, high user immersion (GEQ-Core immersion dimension: 2.45/4), and minimal simulator discomfort (SSQ total score: 5.12/48).

The directional alignment between game performance indicators and B-CATS scores provides preliminary evidence for Fruit Pioneer’s utility as an auxiliary diagnostic tool. Although correlations were modest, directional consistency emerged across core cognitive domains relevant to schizophrenia. Specifically, the executive function demands assessed by TMTA/TMTB (e.g., cognitive flexibility and visual search) are mirrored in the game’s dynamic target prioritization mechanics: participants must suppress impulses toward salient low-value fruits while strategically allocating attention to small, high-reward targets (e.g., apples=3pts) under time constraints—paralleling real-life goal-directed planning (35). Simultaneously, working memory processes indexed by DSST (symbol-digit mapping and processing speed) find direct analogues in the requirement to retain fruit-point associations (e.g., “banana=2pts”) while rapidly executing visually cued motor responses (36). Critically, the bomb inhibition mechanism operationalizes attentional vigilance; avoiding penalty-inducing bombs amidst moving fruits necessitates continuous stimulus discrimination and response control—quantified through BOMB hit rates that negatively correlated with TMT performance. These domain-specific mappings resonate with Kourtesis et al. (2020) (15)but extend prior work by explicitly linking VR mechanics to MATRICS-aligned cognitive constructs through ecologically valid paradigms.

The diagnostic validity of *Fruit Pioneer*, evidenced by AUC values exceeding 0.7 for 13 of 16 indicators, further underscores the potential of VR in cognitive assessment. The composite TGS indicator, which integrates working memory retention, process management, visual search efficiency, and inhibitory control, achieved exceptional classification accuracy with an AUC of 0.911, sensitivity of 83.72%, and specificity of 89.06%. Employing a TGS threshold of ≤569.5 enabled rapid screening within 5 minutes, achieving a case detection rate exceeding 80%, which highlights its clinical utility as a preliminary screening biomarker.

Systematic reviews affirm the safety and acceptability of VR in psychosis populations, with studies reporting positive user attitudes and no increases in anxiety or simulator sickness during or after exposure (31). This established safety context supports the quantitative findings from the *Fruit Pioneer* assessment: the quantitative assessment of adverse effects demonstrates *Fruit Pioneer*’s favorable safety profile for schizophrenia populations. Patients reported minimal negative affect (0.76 ± 0.85) and tension (0.66 ± 0.85). Physiological tolerance was further evidenced by the absence of severe cybersickness, with isolated cases of transient dizziness resolving spontaneously within 15 minutes. Importantly, preserved engagement capacity was observed despite diagnostic status. Patients maintained moderate positive affect scores (2.51 ± 1.04) and immersion levels (2.28 ± 1.11), confirming the tool’s motivational suitability. This safety profile can be attributed to three protocol safeguards: (1) Exclusion of actively psychotic individuals; (2) Emotionally neutral task design avoiding anxiety triggers; (3) Strict 5-minute exposure below symptom provocation thresholds. Systematic reviews confirm that brief VR exposures (<10 minutes) demonstrate optimal safety in vulnerable populations (37), supporting our conservative duration choice. While patients exhibited moderately higher oculomotor discomfort than controls (3.33 ± 3.62 vs. 1.88 ± 2.67, p=0.008), these values remain within acceptable clinical limits. Future iterations may further reduce discomfort through anti-aliasing optimization, though current data validate the protocol’s safety for implementation.

These findings align with advancements in VR cognitive assessment tools (14, 26), such as the VRFCAT (Virtual Reality Functional Capacity Assessment Tool), which assesses functional capacity in schizophrenia through simulated daily tasks (e.g., grocery shopping, bus fare payment). However, VRFCAT’ s reliance on socially contextualized tasks introduces symptom-related confounds: Patients with reduced emotional experience show significantly poorer performance on socially demanding subtasks (r = 0.18, p < 0.05), limiting its utility in populations with prominent negative symptoms (38). Notably, *Fruit Pioneer* addresses critical limitations of existing paradigms through three key design innovations: reducing task complexity by eliminating narrative contexts and focusing solely on the core fruit-cutting task, thereby minimizing cognitive load and dependency on educational background or symptom stability; improving time efficiency through a 5-minute protocol compared to traditional tests requiring 60–90 minutes (19) or even shorter alternatives like B-CATS (28); and optimizing diagnostic performance, as evidenced by TGS’s AUC of 0.911—surpassing comparable tools such as Enhance VR (AUC=0.78) (26).

*Fruit Pioneer* demonstrates transformative potential across multiple clinical applications. Its 5-minute automated protocol could facilitate early risk detection in community-based mental health services, while regular gameplay assessments may enable longitudinal monitoring of antipsychotic treatment efficacy or cognitive rehabilitation progress. Additionally, its language-agnostic visual design reduces cultural and educational biases, making it particularly suitable for multilingual or low-literacy populations. The tool’s compatibility with standard VR hardware further aligns with accessibility-first strategies for resource-limited settings.

This study has several limitations. First, the sample size is relatively small, which may affect the generalizability of the results. Second, the demographic characteristics (e.g., age, education, socioeconomic status) of SZs and HCs in this study were not fully matched, and schizophrenia symptom severity was not quantitatively controlled for in the analyses; although confounding factors were corrected, the results may still be affected to some extent. Third, the applicability of *Fruit Pioneer* to other psychiatric or cognitive disorders remains unexplored. Fourth, the hardware requirement (HTC Vive) may limit accessibility in resource-constrained settings. Fifth, the generalizability of our findings to outpatient or early psychosis populations, who may present with different symptom profiles or acuity, remains unknown. Finally, while the fruit-based design minimizes language barriers, its cultural acceptability across diverse populations requires further validation, as certain fruits or the cutting mechanic may carry unintended connotations in specific cultural contexts. Future studies should validate these findings in larger cohorts, rigorously control for additional confounders (including symptom severity and prior VR exposure), and investigate the tool’s broader clinical relevance. Further research should also explore the relationship between game-derived cognitive assessments and functional outcomes to strengthen ecological validity, and evaluate the feasibility of implementing Fruit Pioneer on lower-cost VR platforms to enhance accessibility.

## 5 Conclusion

This study confirmed that the VR serious game *Fruit Pioneer* can effectively distinguish patients with schizophrenia from healthy people, and its short time, high discriminative validity, and positive gaming experience provide a new ecological tool for clinical cognitive assessment. Although the correlation with traditional tests is weak, its ability to capture reality-based cognitive function compensates for the shortcomings of existing tools. In the future, through technical optimization and cross-center validation, this tool is expected to become an important auxiliary means for cognitive assessment and rehabilitation intervention in schizophrenia.

## Data Availability

The raw data supporting the conclusions of this article will be made available by the authors, without undue reservation.
